# Exosomes derived from CD271^+^CD56^+^ bone marrow mesenchymal stem cell subpopoulation identified by single-cell RNA sequencing promote axon regeneration after spinal cord injury

**DOI:** 10.7150/thno.89008

**Published:** 2024-01-01

**Authors:** Yi Sun, Quanbo Liu, Yiming Qin, Yan Xu, Jinyun Zhao, Yong Xie, Chengjun Li, Tian Qin, Yuxin Jin, Liyuan Jiang, Yong Cao, Hongbin Lu, Jianzhong Hu

**Affiliations:** 1 Department of Spine Surgery and Orthopaedics, Xiangya Hospital, Central South University, Xiangya Road 87, Changsha 410008, China.; 2 Department of Sports Medicine, Xiangya Hospital, Central South University, Xiangya Road 87, Changsha 410008, China.; 3 Key Laboratory of Organ Injury, Aging and Regenerative Medicine of Hunan Province, Xiangya Road 87, Changsha 410008, China.; 4 Hunan Engineering Research Center of Sports and Health, Changsha 410008, China.; 5 National Clinical Research Center for Geriatric Disorders, Xiangya Hospital, Central South University, Xiangya Road 87, Changsha 410008, China.

**Keywords:** Bone Marrow Mesenchymal Stem Cell, Single-Cell RNA Sequencing, Spinal cord injury, Axon Regeneration, Exosome

## Abstract

**Rationale:** Spinal cord injury (SCI) results in neural tissue damage. However, the limited regenerative capacity of adult mammals' axons upon SCI leads to persistent neurological dysfunction. Thus, exploring the pathways that can enhance axon regeneration in injured spinal cord is of great significance.

**Methods:** Through the utilization of single-cell RNA sequencing in this research, a distinct subpopulation of bone marrow mesenchymal stem cells (BMSCs) that exhibits the capacity to facilitate axon regeneration has been discovered. Subsequently, the CD271^+^CD56^+^ BMSCs subpopulation was isolated using flow cytometry, and the exosomes derived from this subpopulation (CD271^+^CD56^+^ BMSC-Exos) were extracted and incorporated into a hydrogel to create a sustained release system. The aim was to investigate the therapeutic effects of CD271^+^CD56^+^ BMSC-Exos and elucidate the underlying mechanisms involved in promoting axon regeneration and neural function recovery.

**Results:** The findings indicate that CD271^+^CD56^+^ BMSC-Exos share similar physical and chemical properties with conventional exosomes. Importantly, in an SCI model, in situ implantation of CD271^+^CD56^+^ BMSC-Exos hydrogel resulted in increased expression of NF and synaptophysin, markers associated with axon regeneration and synapse formation, respectively. This intervention also contributed to improved neural function recovery. In vitro experiments demonstrated that CD271^+^CD56^+^ BMSC-Exos treatment significantly enhanced axon extension distance and increased the number of branches in dorsal root ganglion axons. Moreover, further investigation into the molecular mechanisms underlying CD271^+^CD56^+^ BMSC-Exos-mediated axon regeneration revealed the crucial involvement of the miR-431-3p/RGMA axis.

**Conclusion:** In summary, the implantation of CD271^+^CD56^+^ BMSC-Exos hydrogel presents a promising and effective therapeutic approach for SCI.

## Introduction

Spinal cord injury (SCI) is a permanent injury resulting from direct tissue trauma, leading to the loss of motor, sensory, and autonomic nervous function 1. SCI poses a substantial global health concern, with an annual worldwide incidence exceeding 700,000 reported cases 2. Current treatment strategies for SCI mainly focus on patient stabilization, prevention of complications, and physical rehabilitation 3. Although some progress has been made in clinical management, the recovery of SCI remains limited, emphasizing the urgent need for alternative therapeutic approaches to address paralysis.

The pathogenesis of SCI involves two distinct mechanisms: primary mechanical injury and secondary injury. The initial trauma causes primary damage, including neuronal and glial cell death within minutes to hours, leading to tissue damage and neural necrosis. Secondary injury, triggered by the primary injury, further exacerbates tissue damage and functional loss 4. Adult mammals exhibit limited axon regeneration ability following SCI, resulting in permanent neurological dysfunction 5, 6. Therefore, developing novel treatment strategies to enhance axon regeneration upon SCI remains a crucial unmet demand.

In the past few years, there has been a notable increase in interest surrounding the potential therapeutic application of mesenchymal stem cells (MSCs) for SCI 7-9. Multiple studies have indicated that intravenous infusion of bone marrow-derived MSCs (BMSCs) reduces lesion size, provides neuroprotection, promotes axon sprouting, and improves motor recovery in experimental models of SCI 10-12. However, MSCs are a heterogeneous cell population, and their relatively low purity hinders their broader clinical application 13. Hence, identifying distinct subpopulations of MSCs, understanding their specific functions, and exploring their relationships are vital for interpreting clinical trial outcomes and ultimately enhancing the efficacy of MSC-based therapies. Recently, single-cell RNA sequencing (scRNA-seq) technology has emerged as an effective tool for studying tissue and cellular heterogeneity by profiling the RNA expression of individual cells within a sample 14. This approach enables the identification of cell types, states, functions, and relationships, thus offering an unprecedented opportunity to uncover subpopulations with shared gene expression profiles within heterogeneous cell populations. Although scRNA-seq has revealed the heterogeneity of BMSCs and Wharton's Jelly-derived MSCs, and identified subpopulations with unique functions 15, 16, the presence of subpopulations related to neural regeneration within MSCs remains largely unknown.

Evidence has accumulated to support the notion that the therapeutic effects of MSCs are mediated by the secretion of bioactive components, including cytokines, growth factors, and exosomes 17-19. Research has shown that MSC-derived exosomes, exhibits similar regenerative capabilities to their parent cells and can effectively promote tissue repair 20, 21. Exosomes, a major subpopulation of extracellular vesicles, exhibit advantageous properties such as high biocompatibility and prolonged circulation, making them an ideal choice for enhancing drug delivery and therapeutic efficacy 22, 23. Given the diverse microenvironments and cell sources, exosomes display significant heterogeneity in molecular composition, and their biological and clinical value remains largely unexplored 24. It is widely acknowledged that exosomes comprise distinct subpopulations with diverse biological functions 25. Therefore, investigating the functions of specific exosome subpopulations will offer new insights into cell-free disease treatment.

In this study, we performed scRNA-seq analysis on isolated human CD271^+^ bone marrow mononuclear cells, identifying a unique subpopulation of CD271^+^CD56^+^ MSCs with a demonstrated ability to promote axon regeneration. Using flow cytometry, we isolated the specific CD271^+^CD56^+^ BMSC subpopulations and extracted their exosomes (CD271^+^CD56^+^ BMSC-Exos). Additionally, we engineered CD271^+^CD56^+^ BMSC-Exos hydrogel and investigated its role in axon regeneration and neural functional recovery. Furthermore, we explored the molecular mechanisms underlying the promotion of axon regeneration by CD271^+^CD56^+^ BMSC-Exos and identified the miR-431-3p/Repulsive Guidance Molecule Family Member A (RGMA) axis as a key regulator in this process. In conclusion, our results showed that CD271^+^CD56^+^ BMSC-Exos hydrogel significantly enhance axon regeneration and improve functional recovery following SCI.

## Methods

### Bioinformatic analysis of the single-cell transcriptomic dataset

The single-cell RNA sequencing data of the CD271^+^ bone marrow mononuclear cells from human (GSE147287) and the information on the corresponding annotation were retrieved from the GEO database. CellRanger (v6.1.2) software was used to transform the Illumina output into gene-barcode count matrices for further analysis in our study. Data processing and analysis were performed using the R package "Seurat" (v5.0). Cells having > 6000 genes and > 20% of mitochondrial transcripts were removed. The gene expression matrix was normalized and scaled. We selected the top 50 principal components by performing PCA based on 5000 variable genes. The "harmony" algorithm was applied to correct the batch effect. The FindNeighbors and FindClusters functions were used to cluster cells on a shared-nearest-neighbor graph. Clusters were annotated to each cell type based on markers from previous study. We generated UMAP plots to visualize annotated cell clusters. The markers of each cell type were calculated using the FindAllMarkers function, which compares gene expression between clusters by performing the Wilcoxon rank-sum test. While analyzing the BMSCs subclusters, 6 clusters were identified. Among them, cluster5 with relatively high expression of CD56 were defined as CD56^+^ BMSCs. The Gene Ontology (GO), Kyoto Encyclopedia of Genes and Genomes Pathway-based Enrichment Analysis (KEGG) pathway enrichment, and GSEA of the marker genes were performed as above.

### Mice

The 8-week-old female C57BL/6 mice were procured from the Laboratory Animal Center at Central South University. The handling of animals was conducted in accordance with ethical guidelines approved by the Animal Experiment Ethics Committee of Central South University, adhering to relevant regulations. Throughout the study, mice were provided with unrestricted access to food and water. The mice were kept under constant temperature, humidity, and a 12 h light/dark cycle maintained throughout the year.

### Cell culture

Human bone marrow was obtained from Xiangya Hospital of Central South University with ethical approval from the ethics committee. BMSCs were isolated using density gradient centrifugation. The plasma layer supernatant containing fat cells was discarded. The cells were then seeded at a density of 2 × 10^5^/cm^2^ in Minimum Essential Medium (MEM) containing 15% fetal bovine serum (FBS, CellMax, China) and cultured at 37 °C in a humidified atmosphere containing 5% CO_2_. After adhering, the spindle-shaped cells were cultured until they reached 80% confluence. Subsequently, they were digested using 0.25% trypsin (Gibco, USA) 26.

The dorsal root ganglia (DRG) were obtained from 8-week-old mice 27. The nerve root was removed, and the ganglion was split in half and cultured in neurobasal medium (Gibco, USA) supplemented with 2% B-27 (Gibco, USA), 1% penicillin/streptomycin (Solarbio, China), and nerve growth factor (50 ng/mL, Novoprotein, China). 48-well plate (NEST, China) were pre-treated with poly-L-lysine (Gibco, USA) and laminin (10 mg/mL, Gibco, USA).

### Flow cytometry

Cell sorting was performed as previously reported 28. Following washing, the single-cell suspension was treated with Human BD Fc Block solution (BD, USA) and incubated at 4 °C for 10 min. The harvested cells were stained with Zombie Aqua (Biolegend, USA) for 15 min. Subsequently, they were incubated with specific antibodies (Biolegend, USA) at 4 °C for 30 min, including anti-CD271-APC, anti-CD31-PE, anti-CD45-PE, anti-CD235-PE, anti-CD90-APC/Cy7, anti-CD73-PE/CF594, and anti-CD56-PE/Cy7. After washing, the cells were resuspended in PBS. The experiment was conducted using a FACS Aria II SORP cell sorting instrument (BD, USA) and analyzed using FlowJo software (BD, USA). CD271^+^CD56^+^ BMSCs were cultured in vitro until passage 3 for extraction of exosomes. The information regarding all antibodies used is provided in Table S1.

### Multilineage differentiation analysis

CD271^+^CD56^+^ BMSCs were cultured in osteogenic induction medium, adipogenic induction medium, and chondrogenic induction medium (Haixing Biosciences, China), respectively. Osteoblasts, adipocytes, and chondrocytes were stained with Alizarin red, oil red O, and Alcian blue, respectively.

### Preparation and identification of Exos

The extraction method of exosomes, as previously described, collect the supernatant of cells cultured in Exos free FBS (Inner Mongolia Opcel Biotechnology Co., Ltd, China) for 48 h, and sequentially centrifuging at 500 g for 10 min, 2000 g for 30 min, 10000 g for 60 min, and 100000 g for 60 min to acquire sediment 28. The sediment was then re-suspended in PBS. To observe the morphology of exosomes, transmission electron microscopy (TEM) was utilized. Additionally, the size and concentration of the exosomes were assessed using a flow nanoanalyzer instrument. In vitro, western blot was employed to confirm the presence of CD9, CD63, CD81 and calnexin.

### Preparation and characterization of the hydrogel

Hydrogels were prepared by combining a specific ratio of GelMA and photoinitiators (LAPs), following the synthesis methods described in previous reports 29. For GelMA/LAP hydrogel preparation, lyophilized GelMA was dissolved in PBS at a concentration of 10% (w/v) at 40 ℃. Then, a mixture of 10% GelMA and LAP was prepared to obtain a final concentration of 5% (w/v) GelMA and 0.1% (w/v) LAP 30.

The hydrogel solution was irradiated at 37 ℃ using a UV lamp at 365-405 nm for 30 s, resulting in the transformation of the soluble state to a gel state. The structural and morphological variations of the hydrogels were investigated using a Mira3-TESCAN scanning electron microscope (SEM).

In vitro degradation of the hydrogels was assessed by preparing 100 μL of GelMA hydrogels, with the initial weight recorded as Md_0_. The hydrogels were then immersed in pH 7.4 PBS solution and incubated for 28 days in a shaker at 4 °C. At specific time points (0 days, 1 day, 3 days, 7 days, 10 days, 14 days, 21 days, and 28 days), the supernatant was absorbed, and the weight of the hydrogel was measured and recorded as Md_t_. The degradation rate of the hydrogels was calculated using the formula DR = ((Md_t_-Md_0_)/Md_0_) × 100% 31.

The rheological properties of the hydrogel were determined using a rheometer (Anton Par, Austria) equipped with a temperature control unit using a plate-plate configuration (plate diameter of 20 mm). Frequency sweep tests were performed from 0.1 to 1 Hz at 37 ℃ and 1% strain.

### CD271^+^CD56^+^ BMSC-Exos loading and releasing in vitro

To load exosomes, the exosomes were labeled with the red fluorescent lipophilic dye PKH26 (Sigma Aldrich, USA). Then, the PKH26-labeled CD271^+^CD56^+^ BMSC-Exos suspension (100 μg/μL) was added to the hydrogel solution at a final concentration of 20 mg/mL. Subsequently, 20 μL of the composite was irradiated under UV light to produce a PKH26-labeled CD271^+^CD56^+^ BMSC-Exos hydrogel, which was then prepared for confocal (LSM780, Zessis) multilayer scanning and 3D reconstruction based on scan data.

For the release curve of the CD271^+^CD56^+^ BMSC-Exos hydrogel, 100 μL of the Exos hydrogel (20 mg/mL, after UV irradiation) was immersed in 500 μL of PBS medium at 37 ℃. At regular intervals, the same amount of PBS medium was replenished after collecting 500 μL of the sample. The quantitative determination of exosome release was performed using the Micro BCA^TM^ Protein Assay Kit (ThermoFisher, USA) 32. The release curve was plotted based on the release.

### Determination of Exos uptake in vitro and in vivo

Exosomes were marked with PKH26 and 1,1-octadecyl-3,3,3-tetramethylindole tricarbonylcyanine iodide (DIR) (Sigma Aldrich, USA) and collected by ultracentrifugation. DRG and PKH26-labeled exosomes were cultured for a total of 24 h, and the internalization of exosomes was assessed 33. To track CD271^+^CD56^+^ BMSC-Exos in vivo, PKH26 fluorescently labeled exosomes were mixed with hydrogel and implanted at the lesion site after injury. 14 days after injury, spinal cord tissue was collected, sections were prepared for immunostaining, and the uptake of exosomes by neurons was analyzed. The distribution of fluorescence signals in exosomes immediately after SCI and at 7, 14, 21, and 28 days was assessed using the Xenogenic IVIS imaging system (Caliper Life Sciences, USA).

### Biocompatibility evaluation of hydrogel

The biocompatibility of hydrogel was assessed through in vitro neural stem cells (NSCs) viability assays using the CCK-8 method, and in vivo multi-organ histological examinations were conducted through H&E staining. For the in vitro assessment, NSCs were seeded in a 96-well plate pre-coated with PBS, hydrogel, BMSC-Exos hydrogel, and CD271^+^CD56^+^ BMSC-Exos hydrogel, respectively. After 1, 3, and 5 days of culture, the OD values of NSCs were measured using an enzyme-linked immunosorbent assay reader. For the in vivo assessment, adult female C57BL/6 mice were anesthetized with pentobarbital sodium and carefully subcutaneously implanted with hydrogel with or without exosomes. After 2 months, the mice were euthanized, and their heart, liver, spleen, lungs, and kidneys were collected for histological analysis using H&E staining.

### SCI model

Adult female C57BL/6 mice (8 weeks old) were randomly divided into the following groups: Sham group (simple laminectomy without SCI), SCI group (implantation of a hydrogel without exosomes), BMSC-Exos hydrogel group (implantation of a hydrogel with BMSC-Exos), CD271^+^CD56^+^ BMSC-Exos hydrogel group (implantation of a hydrogel with CD271^+^CD56^+^ BMSC-Exos), and miR-431-3p^IN^-Exos hydrogel group (implantation of a hydrogel with miR-431-3p inhibitor-loaded CD271^+^CD56^+^ BMSC-Exos). The SCI model was established as described above 34. The mice were intraperitoneally injected with 0.3% pentobarbital sodium (60 mg/kg) to induce anesthesia. A mid-back incision was made to discreetly expose the spinal cord after which a T8 laminectomy was conducted. The 1 mm long spinal cord segment was completely incised and excised. Then, 10 μL of hydrogel with or without exosomes was injected locally at the injury site, followed by UV irradiation to solidify the gel. The muscle and skin were sutured sequentially, and manual bladder emptying was performed twice a day for the following two weeks until bladder function was restored.

### Locomotor function assessment

The Basso Mouse Scale (BMS) Hindlimb Movement Scale was used to assess the recovery of hindlimb motor function at specific intervals before and after SCI (1, 3, 7, 14, 21, 28, 35, 42, 49, and 56 days) 35. This scale allows scores to range from 0 (complete paralysis) to 9 (normal hindlimb motor function). Two impartial assessors evaluated the motor function of the hind limbs and determined the average score to obtain the final BMS score.

### Electromyography

Hindlimb motion-evoked potentials (MEPs) were measured 56 days after injury using previously reported methods 36. The mice were anesthetized, and stimulus electrodes were placed on the skull of the cortical motor centers, while recording electrodes were inserted into the contralateral hindlimb muscle. Electrical stimulation was performed at 3 V and 333 Hz, repeating every 2 s. The average amplitude of MEPs 56 days after SCI was recorded.

### MicroRNA array assay

As described above, total RNA is extracted from Exos. After passing the quality and quantity tests, a small RNA sample pre-kit was used to prepare the library. The resulting product was reverse-transcribed to synthesize cDNA, and target DNA fragments were isolated to obtain cDNA libraries. The quality and quantity of the library were carefully evaluated using the Agilent 2100 Bioanalyzer through an automated electrophoresis process. After passing the library check, the library was sorted using Hiseq/Miseq.

### qRT-PCR

Total RNA was extracted from exosomes and DRG using TRIzol reagent (Invitrogen, USA). Reverse transcription of microRNAs was performed using the MicroRNA First Strand cDNA Synthesis Kit (Tailing Reaction, China). qRT-PCR of mRNA and microRNA was performed using the GoTaq qPCR Master Mix Kit (Promega, USA) and the Quantitative PCR System (ABI, USA). GAPDH and U6 were used as internal parameters, and the relative expression level of mRNA or microRNA was evaluated using the 2^-ΔΔCT^ method 37. The qRT-PCR primer sequences used in this study are listed in Table S2.

### Western blot

Proteins from spinal cord tissue and DRG were extracted using RIPA (Solarbio, China) and measured using a BCA kit. Proteins were isolated using 10% SDS-PAGE and subsequently transferred to PVDF membranes by electrophoresis. The membranes were sealed with 5% fat-free milk and incubated overnight with primary antibodies, including anti-CD9, anti-CD63, anti-CD81, anti-calnexin, anti-RGMA, and anti-GAPDH. The membranes were then washed and incubated with HRP-labeled secondary antibodies for 60 min at room temperature. The immune response bands were visualized using enhanced chemiluminescence reagents (New Cell & Molecular Biotech, China). All antibody information is shown in Table S1.

### Histological and immunofluorescence assessment

Following the administration of saline through the left ventricle, the mice were subsequently fixed using 4% paraformaldehyde solution. Spinal cord and bladder segments were then collected and dehydrated to prepare for sectioning. For H&E staining, the spinal cord and bladder segment was embedded in paraffin, and sagittal sections measuring 5 μm in thickness were stained accordingly. The injury area of the spinal cord and thickness of the muscular layer of the bladder were outlined manually and calculated using ImageJ.

To perform immunofluorescence staining, the spinal cord segments were sectioned into sections measuring 16 μm in thickness 38. These sections were then subjected to an overnight incubation with primary antibodies, namely anti-NF, anti-GFAP, anti-NeuN, and anti-Synaptophysin. Following a thorough washing process, the sections were incubated with secondary antibodies and subsequently stained with DAPI (Gene Tex, USA) to visualize the nuclei. The captured images were acquired using a fluorescence microscope from Zeiss, Germany. Comprehensive information regarding the antibodies used can be found in Table S1.

### MicroRNA in situ hybridization

Fluorescent microRNA in situ hybridization was performed using a miR-431-3p probe (Genechem, China). The sections were deparaffinized, rehydrated, and treated with protease K (20 μg/mL, Ambion, USA) prior to pre-hybridization at 37 °C for 1 h. After pre-hybridization, the sections were incubated with the miR-431-3p probe at 60 °C overnight. Subsequently, the sections were sealed with blocking buffer, incubated with streptavidin, and stained with an anti-NeuN antibody. The relative immunofluorescence intensity ratio of miR-431-3p/NeuN was evaluated using ImageJ.

### Luciferase reporter gene testing

The RGMA plasmid and miR-431-3p mimic were constructed by Genechem. Wild-type RGMA and mutated binding sequences containing miR-431-3p binding sites were cloned into plasmids (Han Biologics, China), named RGMA-WT and RGMA-MUT, respectively. The human adrenal epithelial cell line (293T cells) was co-transfected with luciferase reporter constructs (RGMA-WT or RGMA-MUT) and microRNAs (miR-431-3p-NC or miR-431-3p-mimic). After 2 days, the binding relationship between RGMA and miR-431-3p was detected using the Dual-Luciferase Reporter Gene Detection Kit (Biotim China) according to the instructions.

### Statistical analysis

Statistical analysis was performed using GraphPad Prism 9.0.2 software (GraphPad Software, USA). The data are presented as mean ± standard deviation (SD). The two-tailed Student's t-test was used for two-group comparisons, and one-way ANOVA or two-way ANOVA followed by the Tukey Multiple Comparison Test was used for multiple comparisons. A p value of less than 0.05 (P < 0.05) was considered statistically significant.

## Results

### Identification of BMSC subpopulations promoting axon regeneration by single-cell RNA sequencing

**Figure 1:**
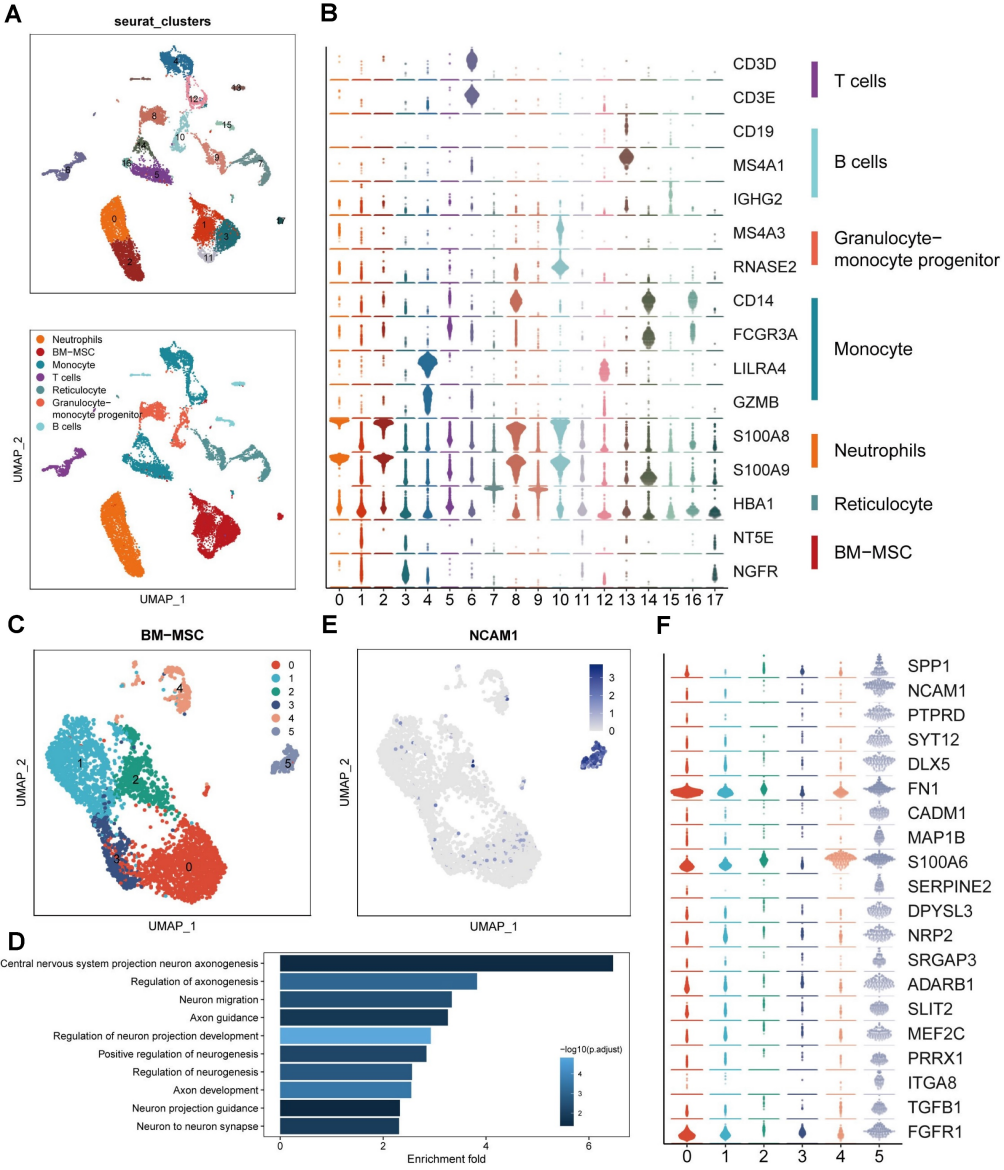
** Identification of BMSC subpopulations promoting axon regeneration by single-cell RNA sequencing. (A)** UMAP plot showing clusters and cell type annotations. **(B)** The expression level of markers of cell type annotations in different clusters. **(C)** UMAP plot showing clusters and BMSCs annotations. **(D)** Enriched GO terms associated with neural regeneration in cluster 5. Bar chart shows the number of genes enriched in each term. Color indicates the adjust p values. **(E)** UMAP plot showing expression levels of NCAM in different clusters. **(F)** The expression level of markers of enriched GO terms associated with neural regeneration in different clusters.

To study the transcriptome diversity of BMSCs, we performed bioinformatics analysis on scRNA-seq data obtained from CD271^+^ bone marrow mononuclear cells isolated from human bone marrow. Through graph clustering, we divided the cells into 18 different clusters (clusters 0-17) (Figure 1A). Clusters 1, 3, 11, and 17 exhibited high expression levels of BMSC marker genes, including NT5E (CD73) and NGFR (CD271). We observed the following cell types based on marker expression: 1) Clusters 0 and 2 were neutrophils expressing high levels of S100A8 and S100A9; 2) Clusters 4, 5, 12, 14, and 16 were monocytes expressing high levels of CD14; 3) Clusters 13 and 15 were B cells expressing CD19, MS4A1, and IGHG2; 4) Cluster 6 was a T cell expressing CD3; 5) Clusters 8 and 10 were granulocyte-monocyte progenitors expressing MS4A3 and RNASE2; and 6) Clusters 7 and 9 were reticulocytes expressing HBA1 (Figure 1B). These findings align with previous reports indicating that CD271^+^ bone marrow mononuclear cells are heterogeneous cell populations comprising multiple cell types 15. To explore the cell heterogeneity of BMSCs, we extracted cells expressing high levels of NT5E and NGFR for further analysis, resulting in the division of BMSCs into 6 clusters through reclustering analysis (Figure 1C).

In order to explore the variances in biological processes among subclusters of BMSCs, we conducted functional enrichment analysis using GO and KEGG. This analysis focused on the identification of differentially expressed genes within each cluster. Cluster 5 exhibited enrichment of several pathways related to neural regeneration, including "Central nervous system projection neuron axonogenesis," "Axon guidance," and "Axon development," among others (Figure 1D). These results suggest that cluster 5 may possess potential functions in promoting axon regeneration. Additionally, we identified that neural cell adhesion molecule (NCAM, CD56) was highly expressed and specific to cluster 5, serving as a marker for this cluster (Figure 1E). Moreover, we examined the genes enriched in these biological processes and identified 20 genes that regulate neural regeneration, including SPP1, SLIT2, TGFB1, and SERPINE2, with significantly higher expression levels in cluster 5 (Figure 1F). Based on the results of single-cell RNA sequencing analysis, we identified cluster 5 (CD271^+^CD56^+^ BMSCs) as the target for further research.

### Acquisition and identification of CD271^+^CD56^+^ BMSC-Exos

**Figure 2:**
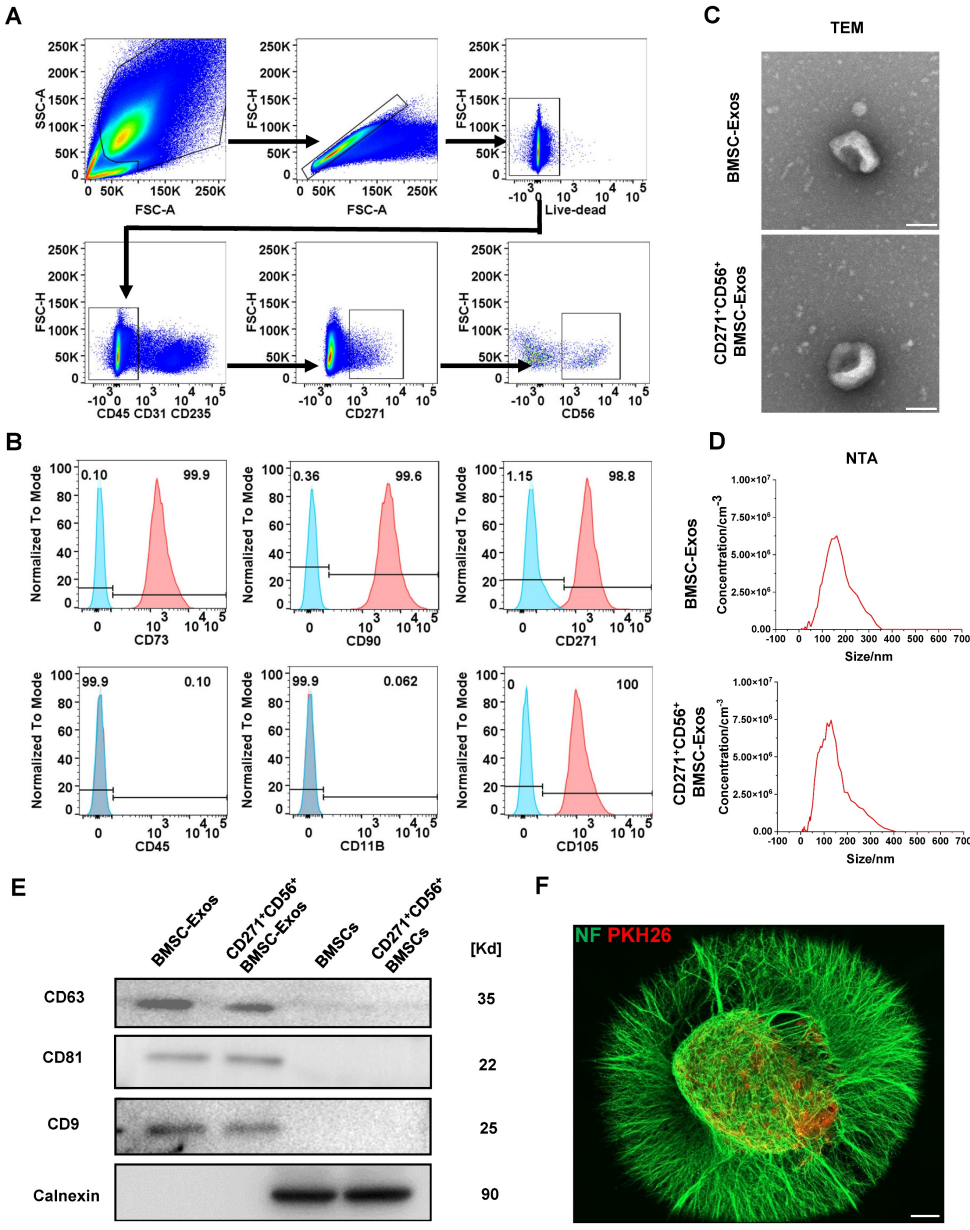
** Acquisition and identification of CD271^+^CD56^+^ BMSC-Exos. (A)** Flow cytometry sorting of live CD271^+^CD56^+^ BMSCs. **(B)** Flow cytometry analysis of CD271^+^ CD56^+^ BMSCs cell markers. The blank control is shown as a blue dashed curve and the test sample is shown as a red curve. **(C)** Transmission electron microscopy (TEM) image of CD271^+^CD56^+^ BMSC-Exos. Scale bar: 100 nm. **(D)** NanoSight Tracking Analysis (NTA) of CD271^+^CD56^+^ BMSC-Exos. **(E)** Western blot analysis of the Exos markers. **(F)** Representative immunofluorescence image of DRG (NF marker, green) uptake Exos (PKH26 marker, red). Scale bar, 200 μm.

Flow cytometry was utilized to isolate CD271^+^CD56^+^ BMSCs from human bone marrow. The sorted cells were confirmed as live cells (negative for Zombie) and tested negative for CD31, CD45, and CD235, indicating the absence of endothelial, immune, and blood cells. Additionally, the cells exhibited positive expression of CD271, confirming the presence of MSCs, and positive expression of CD56, indicating the presence of CD271^+^CD56^+^ subpopulations (Figure 2A). The identification of CD271^+^CD56^+^ BMSCs was performed at passage 3, demonstrating that over 99% of the cells expressed CD90 and CD73, while less than 1% expressed CD11b and CD45. Moreover, the positive rates of CD56 and CD271 were both above 90%, indicating that the CD271^+^CD56^+^ subpopulations maintained their distinctive features even after being cultured (Figure 2B). CD271^+^CD56^+^ BMSCs exhibited a fibroblast-like morphology in the light field image. The results of multilineage differentiation confirmed that CD271^+^CD56^+^ BMSCs can differentiate into adipocytes, osteocytes, and chondrocytes, thereby supporting their stem cell properties (Figure S1).

Subsequently, BMSC-Exos and CD271^+^CD56^+^ BMSC-Exos were isolated for TEM, NTA, and Western blot. TEM images indicated a similar cup-shaped morphology of Exos in both groups, and NTA results showed a comparable size distribution of nanoparticles (Figure 2C-D). Western blot analysis demonstrated a notable enrichment of CD81, CD63, and CD9 in both BMSC-Exos and CD271^+^CD56^+^BMSC-Exos, while the expression of calnexin was nearly absent (Figure 2E). Furthermore, to investigate the uptake of CD271^+^CD56^+^ BMSC-Exos by DRG, PKH26-labeled CD271^+^CD56^+^ BMSC-Exos were co-cultured with DRG. After 24 h of incubation, immunofluorescence staining revealed the internalization of PKH26-labeled Exos into the DRG (Figure 2F).

### Characterization of CD271^+^CD56^+^ BMSC-Exos hydrogel

**Figure 3:**
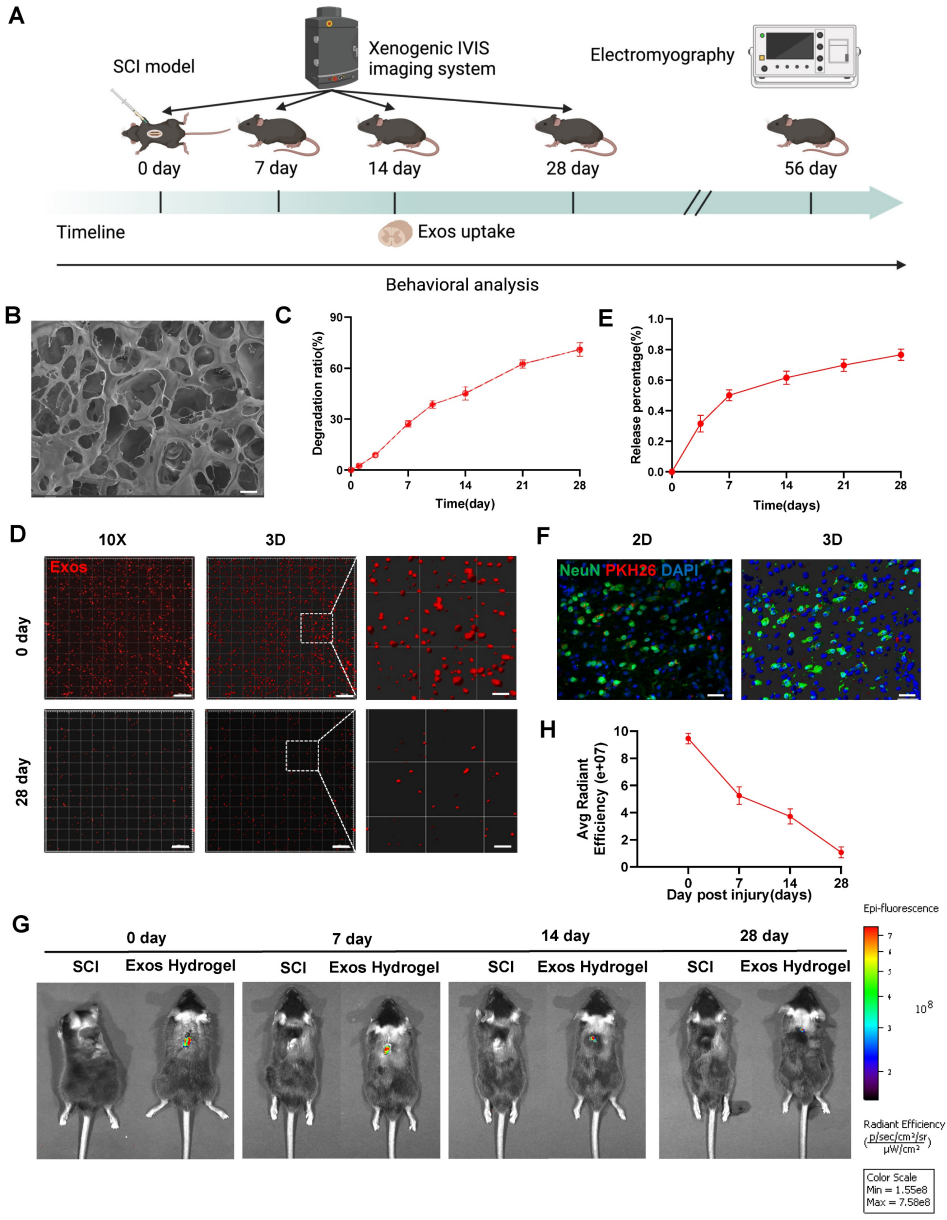
** Characterization of CD271^+^CD56^+^ BMSC-Exos hydrogel. (A)** Schematic diagram of the experimental procedure. **(B)** Mira3-TESCAN scanning electron microscope (SEM) images showed the microstructures of the hydrogel. Scale bar: 20 μm. **(C)** In vitro degradation curves of hydrogels over time based on gravimetric measurements. **(D)** Confocal multilayer scanning and 3D reconstruction of PKH26 markers retained within the hydrogel. Scale bar, 20 μm, Scale bar, 4 μm (enlarged view). **(E)** Release curves of Exos at different time points. n = 6. **(F)** Representative immunofluorescence image of uptake of Exos (PKH26 marker, red) by neurons (NeuN marker, green), 14 dpi. The nucleus is counterstained with DAPI (blue). Scale bar, 40 μm. **(G)** In vivo tracking of DiR-labeled Exos in the injured spinal cord after 0, 7, 14, and 28 days of injury center hydrogel mixed exosome administration. Each group n = 6. **(H)** Quantification of (G). Data are expressed as mean ± SD. (One-way ANOVA between multiple groups plus Tukey's hoc test).

Figure 3A presents a schematic diagram illustrating the experimental process of Exos uptake and tracing. Subsequently, we developed hydrogel encapsulating Exos to ensure their stable release. Figure 3B demonstrates that the Exos hydrogel exhibits favorable pore morphology with uniform pore wall thickness, a smooth surface, and good connectivity. Degradation curve analysis indicated that the Exos hydrogel degraded by more than 60% after 28 days (Figure 3C). As illustrated in Figure S2, the storage modulus (G′) of hydrogels, with and without the loading of CD271^+^CD56^+^ BMSC-Exos, at 1 Hz are 615.0 Pa and 620.9 Pa, respectively. The loss modulus (G″) are 108.0 Pa and 122.4 Pa for the two cases, respectively. Notably, the values of G′ are consistently higher than those of G″, indicating that these hydrogels exhibit viscoelastic solid properties. Furthermore, it is noteworthy that these hydrogels modulus values fall within the reported range of spinal cord tissue modulus, typically ranging from 100 to 3000 Pa 39. The CCK-8 assay results from Figure S3 demonstrate that even after 5 days of co-cultivation, hydrogel did not affect the viability of NSCs. Additionally, our findings show that implantation of hydrogel did not induce appreciable cardiotoxicity, hepatotoxicity, splenic cytotoxicity, pulmonary toxicity, or nephrotoxicity, as showed in Figure S4. These results collectively suggest that the hydrogel exhibits good biocompatibility.

To assess the release and internalization of Exos, we employed confocal scanning to observe the PKH26-labeled Exos retained within the hydrogel. The results revealed an even distribution of Exos throughout the hydrogel (Figure 3D). The release curve demonstrated a continuous release of loaded Exos from the hydrogel, reaching approximately 80% of the cumulative amount after 28 days (Figure 3E). To validate the in vivo therapeutic efficacy, PKH26-labeled Exos hydrogel were implanted at the site of SCI mice, and red fluorescence signals were detected in neurons after 14 days of SCI (Figure 3F). Additionally, the spatial and temporal distribution of DiR-labeled Exos in vivo was observed using the IVIS imaging system. The results confirmed that the residence time of standalone exosomes in the spinal cord was relatively short, with a significant decrease in fluorescence observed after 3 days of administration. By the 7th day, fluorescence was nearly undetectable, suggesting a potential association with the mobility of exosomes. However, the Exos hydrogel group still exhibited detectable fluorescence signals 28 days after administration. This indicates that the combination of hydrogel and Exos effectively achieves long-term sustained release, addressing the issue of Exos mobility and retention in vivo (Figure 3G-H and Figure S5).

### CD271^+^CD56^+^ BMSC-Exos improve functional recovery after SCI and enhance axon regeneration in vivo and in vitro

**Figure 4:**
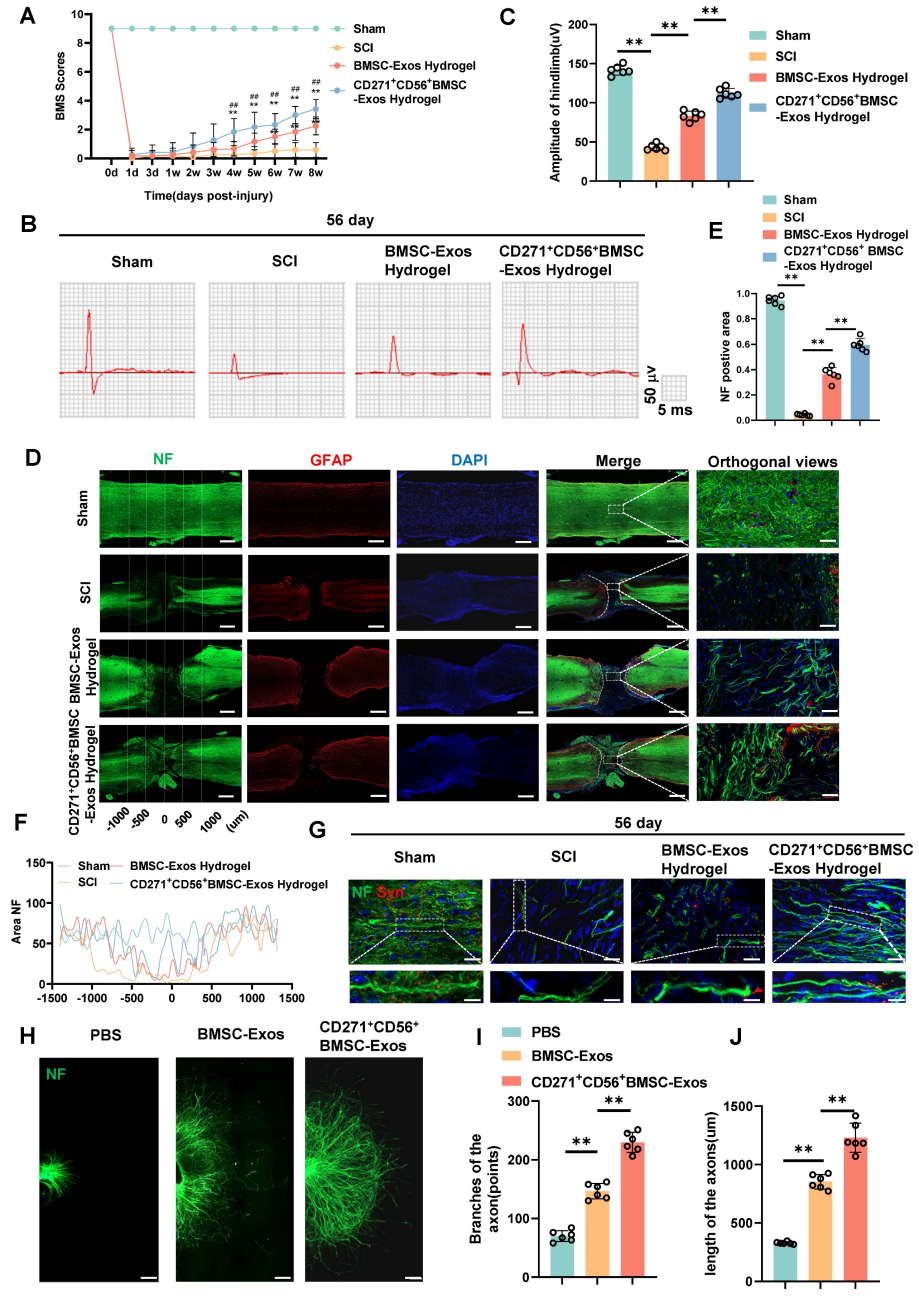
** CD271^+^CD56^+^ BMSC-Exos improve functional recovery after SCI and enhance axon regeneration in vivo and in vitro. (A)** BMS scores at different time points after SCI in the treatment groups of Sham, SCI, BMSC-Exos, and CD271^+^CD56^+^ BMSC-Exos. Each group n = 12. The data was shown as a mean ± SD, *, # P < 0.05, ##, ** P < 0.01. (Two-way ANOVA plus Tukey'S hoc test between multiple groups). **(B)** At 56 days post-SCI, representative electrophysiological imaging images were recorded for each group. **(C)** Measurement of MEP amplitude in (B). Each group n = 6. **(D)** Representative images of NF (green-Alexa Fluor® 488) immunostained neurons and GFAP (red-Alexa Fluor® 594) astrocytes in mice treated with hydrogel, BMSC-Exos hydrogel and CD271^+^CD56^+^ BMSC-Exos hydrogel after spinal cord injury. Scale bar, 500 μm, Scale bar, 50 μm (enlarge view). **(E)** Quantification of labeled neurons in different groups. Each group n = 6. **(F)** Curves showing the continuous distribution of NF positive neuronal fiber area in (D). **(G)** Representative images of NF (green Alexa Fluor® 488) immunostained neurons and synaptophysin (red Alexa Fluor®594) immunostained neuronal synaptic proteins in mice treated with hydrogel, BMSC-Exos hydrogel and CD271^+^CD56^+^ BMSC-Exos hydrogel after spinal cord injury. Scale bar, 30 μm, Scale bar, 10 μm (enlarge view).** (H)** Representative images of length of the axons and branches of the axons treated with PBS, BMSC-Exos and CD271^+^CD56^+^ BMSCs-Exos in DRG. Scale bar 200 μm. **(I)** Quantification of branches of the axons are shown in (H), n = 6.** (J)** Quantification results for length of the axons are shown in (H), n = 6. The data was shown as a mean ± SD, * P < 0.05, ** P < 0.01. (One-way ANOVA between multiple groups plus Tukey's hoc test).

In this research, we established a model of spinal cord transection to assess the therapeutic efficacy of BMSC-Exos hydrogel and CD271^+^CD56^+^ BMSC-Exos hydrogel in promoting functional recovery following SCI. Prior to the operation and at various time points (1d, 3d, 1w, 2w, 3w, 4w, 5w, 6w, 7w, and 8w) following SCI, we evaluated the BMS score. All groups exhibited motor dysfunction after SCI; however, the BMSC-Exos hydrogel group demonstrated an improvement compared to the SCI group (Figure 4A). Furthermore, the CD271^+^CD56^+^ BMSC-Exos hydrogel group exhibited superior functional recovery compared to the BMSC-Exos hydrogel group. Additionally, we performed electrophysiological analysis of MEPs. At 56 days post-injury, the findings revealed that the amplitude of MEPs in the BMSC-Exos hydrogel group exhibited a notable increase compared to the SCI group. Moreover, the mice treated with CD271^+^CD56^+^ BMSC-Exos hydrogel displayed a higher MEPs amplitude compared to those treated with BMSC-Exos hydrogel (Figure 4B-C). After 56 days post-SCI, tissue continuity and lesion cavity area were assessed through H&E staining (Figure S6A). Quantitative analysis revealed that the CD271^+^CD56^+^ BMSC-Exos hydrogel group exhibited the smallest lesion cavity area when compared to both the BMSC-Exos hydrogel group and the SCI group (Figure S6B). These data provide evidence that the integration of CD271^+^CD56^+^ BMSC-Exos hydrogel within the transected spinal cord is highly efficient, leading to a significant reduction in cavity formation. Furthermore, bladder function was evaluated in aforementioned groups, and the results that were consistent with histological assessments (Figure S7). In summary, CD271^+^CD56^+^ BMSC-Exos hydrogel demonstrated remarkable functional recovery when compared to BMSC-Exos hydrogel.

To explore the role of Exos in axon regeneration after SCI in vivo, we conducted immunofluorescence analysis. The results demonstrated that treatment with BMSC-Exos hydrogel and CD271^+^CD56^+^ BMSC-Exos hydrogel effectively increased the NF^+^ region at the injury site after 56 days of SCI, with CD271^+^CD56^+^ BMSC-Exos hydrogel exerting a stronger effect (Figure 4D-F). Additionally, we performed fluorescence co-staining of NF and synaptophysin to evaluate the synapse formation ability of regenerated axons. The CD271^+^CD56^+^ BMSC-Exos hydrogel group exhibited higher synaptophysin expression compared to the BMSC-Exos hydrogel group (Figure 4G).

Furthermore, we cultured DRG as an in vitro model and labeled axons with anti-NF antibody for immunofluorescence analysis. As reported in previous studies, we measured the maximum extension distance of axons and the number of branches using a fluorescence microscope (Figure S8) 40. The results presented in Figure 4H-J demonstrated that compared to the BMSC-Exos group, the CD271^+^CD56^+^ BMSC-Exos group exhibited longer maximum extension distances of DRG axons and a greater number of axon branches. These findings indicate that CD271^+^CD56^+^ BMSC-Exos possess a stronger ability to enhance axon regeneration than BMSC-Exos, as demonstrated by both in vivo and in vitro experiments.

### miR-431-3p mediates the effects of CD271^+^CD56^+^ BMSC-Exos on improving functional recovery and axon regeneration after SCI

**Figure 5:**
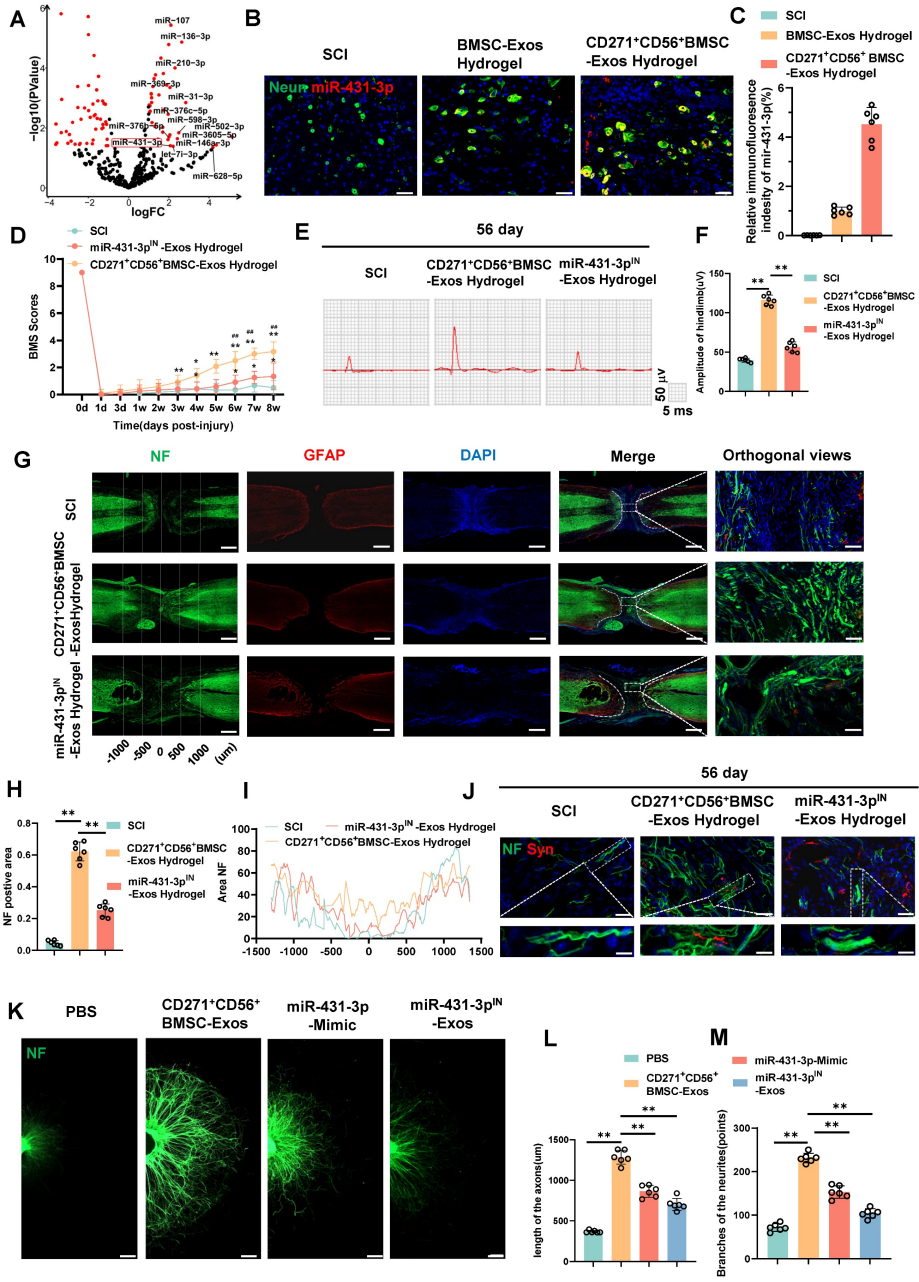
** miR-431-3p mediates the effects of CD271^+^CD56^+^ BMSC-Exos on improving functional recovery and axon regeneration after SCI. (A)** Volcano plot of miRNA expressed differently between CD271^+^CD56^+^ BMSC-Exos and BMSC-Exos. **(B)** Representative images of miR-431-3p in situ hybridized fluorescence of mouse spinal cord tissue treated with BMSC-Exos hydrogel and CD271^+^CD56^+^ BMSC-Exos hydrogel after SCI. Neurons (green), miR-431-3p (red), scale bar, 40 μm. **(C)** Quantification of miR-431-3p immunofluorescence intensity in (B). Each group n = 6.** (D)** BMS scores at different time points after SCI in the treatment groups of SCI, CD271^+^CD56^+^ BMSC-Exos hydrogel and miR-431-3p^IN^-Exos hydrogel. Each group n = 12. The data was shown as a mean ± SD, *, # P < 0.05, ##, ** P < 0.01. (Two-way ANOVA plus Tukey'S hoc test between multiple groups).** (E)** At 56 days post-SCI, representative electrophysiological imaging images were recorded for each group. **(F)** Measurement of MEP amplitude in (E). Each group n = 6. **(G)** Representative images of NF (green-Alexa Fluor® 488) immunostained neurons and GFAP (red-Alexa Fluor®594) astrocytes in mice treated with hydrogel, CD271^+^CD56^+^ BMSC-Exos hydrogel and miR-431-3p^IN^-Exos hydrogel after SCI. Scale bar, 500 μm. Scale bar, 50 μm (enlarge view). **(H)** Quantification of labeled neurons in different groups. Each group n = 6. **(I)** Curves showing the continuous distribution of NF positive neuronal fiber area in (G). **(J)** Representative images of NF (green Alexa Fluor® 488) immunostained neurons and synaptophysin (red Alexa Fluor®594) immunostained neuronal synaptic proteins in mice treated with hydrogel, CD271^+^CD56^+^ BMSC-Exos hydrogel and miR-431-3p^IN^-Exos hydrogel after SCI. Scale bar 30 μm, Scale bar, 10 μm (enlarge view). **(K)** Representative images of length of the axons and branches of the axons treated with PBS, CD271^+^CD56^+^ BMSC-Exos, miR-27a-3p mimic and miR-431-3p^IN^-Exos in DRG. Scale bar 200 μm. **(L)** Quantification results for length of the axons are shown in (K), n = 6. **(M)** Quantification of branches of the axons are shown in (K), n = 6. The data was shown as a mean ± SD, * P < 0.05, ** P < 0.01. (One-way ANOVA between multiple groups plus Tukey's hoc test).

To identify potential molecules that mediate axon regeneration, we performed microRNA sequencing on CD271^+^CD56^+^ BMSC-Exos after extracting total RNA. A total of 61 differentially expressed microRNAs were identified (Figure 5A). We employed the miRNA target databases, miRanda and TargetScan, to determine the target genes of these microRNAs. Subsequently, we performed GO and KEGG analyses on the upregulated microRNAs' target genes, revealing their close association with neural regeneration and their significant involvement in critical processes, particularly axon development (Figure S9). These findings align with the results of single-cell RNA sequencing analysis, indicating the enhanced potential of CD271^+^CD56^+^ BMSC-Exos in promoting the neural functional recovery.

We further quantified the expression levels of 14 highly expressed microRNAs using qRT-PCR (Figure S10). Among them, miR-431-3p, miR-369-3p, miR-376c-5p, miR-376b-5p, miR-136-3p, miR-31-3p, and miR-502-3p were found to be highly expressed in CD271^+^CD56^+^ BMSC-Exos. Notably, miR-431-3p has been reported to be closely related to axon regeneration, exhibiting the most significant differential expression. Consequently, miR-431-3p was selected as the target molecule for subsequent experiments. To investigate whether CD271^+^CD56^+^ BMSC-Exos hydrogel can transfer miR-431-3p to neurons, we performed FISH testing on SCI mice treated with Exos hydrogel. The results confirmed that miR-431-3p is primarily expressed in neurons, and its expression was significantly increased in neurons of the CD271^+^CD56^+^ BMSC-Exos hydrogel group compared to the BMSC-Exos hydrogel group (Figure 5B-C). Thus, these results indicate that CD271^+^CD56^+^ BMSC-Exos hydrogel can transfer miR-431-3p to neurons and upregulate its expression.

To further elucidate the effect of miR-431-3p on mediating the ability of CD271^+^CD56^+^ BMSC-Exos to promote functional recovery after SCI, we generated CD271^+^CD56^+^ BMSCs with miR-431-3p knockdown using miR-431-3p inhibitors. We isolated Exos from both CD271^+^CD56^+^BMSCs and miR-431-3p knockdown CD271^+^CD56^+^ BMSCs. The successful construction of miR-431-3p inhibitor-loaded CD271^+^CD56^+^ BMSC-Exos was verified through qRT-PCR (Figure S11). Subsequently, we implanted hydrogel, CD271^+^CD56^+^ BMSC-Exos hydrogel, and miR-431-3p inhibitor-loaded CD271^+^CD56^+^ BMSC-Exos hydrogel in SCI models to explore the impact of miR-431-3p downregulation on SCI treatment. As depicted in Figure 5D, the beneficial functional effects of CD271^+^CD56^+^ BMSC-Exos hydrogel administration were nullified after miR-431-3p knockdown. Electrophysiological analysis revealed that mice treated with miR-431-3p inhibitor-loaded CD271^+^CD56^+^ BMSC-Exos hydrogel had lower amplitudes compared to those treated with CD271^+^CD56^+^ BMSC-Exos hydrogel (Figure 5E-F). H&E staining revealed that the downregulation of miR-431-3p attenuated the beneficial effects of CD271^+^CD56^+^ BMSC-Exos hydrogel on the lesion cavity area and bladder function in SCI mice (Figure S12-13). In conclusion, these findings suggest that miR-431-3p mediates the role of CD271^+^CD56^+^ BMSC-Exos in promoting functional recovery after SCI.

To elucidate the microRNAs responsible for mediating the biological function of CD271^+^CD56^+^ BMSC-Exos in enhancing axon regeneration, we investigated the role of miR-431-3p in regulating axon regeneration in vivo. As shown in Figure 5G-I, the miR-431-3p inhibitor-loaded CD271^+^CD56^+^ BMSC-Exos hydrogel group exhibited a decreased NF^+^ region compared to the CD271^+^CD56^+^ BMSC-Exos hydrogel group. Additionally, the expression of synaptophysin decreased due to the knockdown of miR-431-3p in Exos (Figure 5J). Subsequently, we administered miR-431-3p mimics and miR-431-3p inhibitors to DRG. These results confirmed that the miR-431-3p mimics increased the extension distance and number of branches of DRG axons, although this effect was not as potent as CD271^+^CD56^+^ BMSC-Exos. Furthermore, miR-431-3p inhibitors reversed the effect of CD271^+^CD56^+^ BMSC-Exos on DRG axons (Figure 5K-M). Therefore, we speculate that CD271^+^CD56^+^ BMSC-Exos contain numerous biomolecules that regulate axon regeneration, with miR-431-3p being a key molecule in this process but not the sole determinant. Overall, these results highlight the essential role of miR-431-3p in enhancing axon regeneration mediated by CD271^+^CD56^+^ BMSC-Exos.

### RGMA overexpression counteracts the beneficial effect of CD271^+^CD56^+^ BMSC-Exos on axon regeneration in vitro

**Figure 6:**
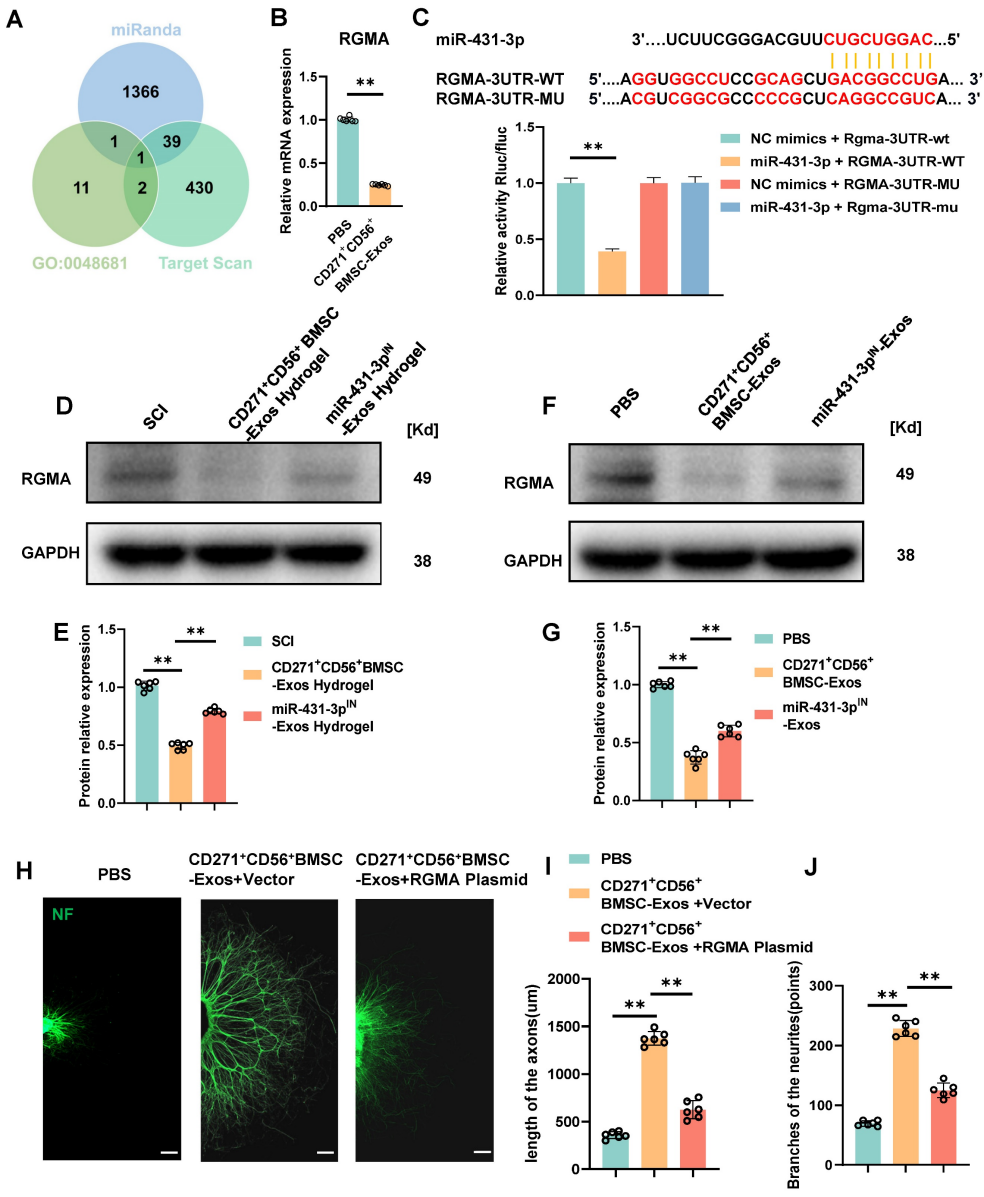
** RGMA overexpression counteracts the beneficial effect of CD271^+^CD56^+^ BMSC-Exos on axon regeneration in vitro. (A)** Venn diagram shows the potential target gene for miR-431-3p. **(B)** Quantification of RGMA of the target gene miR-431-3p in DRG treated with PBS or CD271^+^CD56^+^ BMSC-Exos. Each group n = 6. **(C)** Complementary sequences between miR-431-3p and RGMA's 3' UTR, and relative luciferase activity in the RGMA wild-type (WT) + negative control (NC) group, RGMA-WT + miR-431-3p group, RGMA-MUT + NC group, and RGMA-MUT + miR-431-3p group, n = 3. **(D)**Western blot of RGMA in injured spinal cord tissues treated with hydrogel, CD271^+^CD56^+^ BMSC-Exos hydrogel, and miR-431-3p^IN^-Exos hydrogel. Each group n = 6. **(E)** Quantification of RGMA relative protein expression between different groups in (D). **(F)** Western blot of RGMA in DRG in the PBS, CD271^+^CD56^+^ BMSC-Exos, and miR-431-3p^IN^-Exos group. Each group n = 6. **(G)** Quantification of RGMA relative protein expression in (F).** (H)** Representative images of length of the axons and branches of the axons treated with PBS, CD271^+^CD56^+^ BMSC-Exos + Vector, and CD271^+^CD56^+^ BMSC-Exos + RGMA Plasmid in DRG. Scale bar 200 μm. **(I)** Quantification results for length of the axons are shown in (H).** (J)** Quantification of branches of the axons is shown at the scale shown in (H). Data are expressed as mean ± SD * P < 0.05, ** P < 0.01. (One-way ANOVA between multiple groups plus Tukey's hoc test).

Subsequent exploration was carried out to delve into the potential mechanism underlying the action of miR-431-3p originating from CD271^+^CD56^+^ BMSC-Exos. Based on the online databases and the GO term (GO-0048681, negative regulation of axon regeneration), RGMA was identified as a potential target gene associated with the suppression of axon regeneration (Figure 6A). In order to validate this discovery, the administration of CD271^+^CD56^+^ BMSC-Exos resulted in a significant reduction in the expression level of RGMA in the DRG (Figure 6B). Luciferase reporter experiments were conducted to validate whether RGMA was a direct target of miR-431-3p. Wild-type (WT) and mutant (MUT) sequences of RGMA were constructed. The findings exhibited that miR-431-3p led to a reduction in luciferase activity of the WT construct, whereas there was no change observed in the luciferase activity of the MUT 3'UTR reporter construct. (Figure 6C). These findings provide evidence that miR-431-3p has the capability to directly bind to the 3'UTR of RGMA mRNA, resulting in the inhibition of its expression. Subsequently, CD271^+^CD56^+^ BMSC-Exos hydrogel and miR-431-3p inhibitor-loaded CD271^+^CD56^+^ BMSC-Exos hydrogel were implanted in SCI mice to elucidate the role of miR-431-3p in regulating RGMA expression. Compared to the SCI group, the CD271^+^CD56^+^ BMSC-Exos hydrogel group exhibited lower RGMA expression levels, which were reversed by miR-431-3p knockdown (Figure 6D-E). Similar results were observed in in vitro experiments as well (Figure 6F-G).

To clarify the effect of RGMA in promoting axon regeneration in CD271^+^CD56^+^ BMSC-Exos, we overexpressed RGMA in DRG using an RGMA plasmid. Immunofluorescence experiments revealed that RGMA attenuated the beneficial effect of CD271^+^CD56^+^ BMSC-Exos on axon regeneration (Figure 6H-J). Overall, our findings suggest that miR-431-3p, derived from CD271^+^CD56^+^ BMSC-Exos, enhances axon regeneration by targeting RGMA.

## Discussion

In this study, we identified a subpopulation of BMSCs with axon regeneration-promoting function, termed CD271^+^CD56^+^ BMSCs, through single-cell RNA sequencing analysis. Subsequently, we isolated this subgroup of BMSCs using flow cytometry and extracted their exosomes. Our findings indicate that CD271^+^CD56^+^ BMSC-Exos are more effective in repairing SCI compared to BMSC-Exos. Through a series of experiments, we demonstrated that combining CD271^+^CD56^+^ BMSC-Exos with hydrogel implantation at the injury site can effectively deliver RGMA down-regulation in neurons expressing miR-431-3p. This inhibition of RGMA by miR-431-3p promotes axon regeneration and improves functional recovery, suggesting that CD271^+^CD56^+^ BMSC-Exos hydrogel have potential as a new cell-free therapy for SCI.

Numerous strategies for neural regeneration have been investigated as potential therapies for SCI. Previous studies have shown that BMSC-based treatments hold promise for promoting recovery after SCI 41, 42. However, the low survival rate of transplanted cells in the hypoxic environment surrounding the injury site limits their application in SCI repair. Additionally, heterogeneity in donor conditions, cell type, and differentiation abilities hinders the effectiveness of BMSC-based therapies 7-9. In recent times, exosomes have garnered significant attention as a promising regenerative medicine resource to address these limitations 22. Research has demonstrated that extracellular vesicles isolated from various stem cells yield comparable functional outcomes, making them a viable alternative to BMSC-based therapies 23.

While heterogeneity and differences among MSCs have been considered obstacles to their clinical translation as repeatable, predictable, and standardized treatments, exploration of specific bone marrow MSC subpopulations remains limited 13. Therefore, identifying subpopulations with distinct functions and applying them appropriately may present a novel approach for tissue regeneration.

In this study, we employed single-cell RNA sequencing technology to identify a subpopulation of CD271^+^CD56^+^ BMSCs, which possess the potential to promote axon regeneration. CD271, also known as the low-affinity neurotrophic factor receptor, is highly expressed in the central and peripheral nervous systems. CD271 has been widely used as a marker for isolating BMSCs, effectively eliminating endothelial and hematopoietic cells from the BMSC population 15. CD56 is a calcium-independent binding protein involved in neuronal migration, neurite growth, and synaptic formation. Due to its initial discovery in neural tissues, CD56 is often considered a hallmark of neural lineage commitment 43. Wang et al. 15 demonstrated that the BMSC subpopulation expressing CD56 exhibits good chondrogenic and myogenic potential. Our study revealed enrichment of multiple neural regeneration-related terms in CD271^+^CD56^+^ BMSCs through functional enrichment analysis. Furthermore, the microRNA sequencing results of exosomes derived from CD271^+^CD56^+^ BMSCs also corroborated the enrichment of functions associated with neural regeneration, displaying results consistent with single-cell RNA data. Subsequently, we conducted a series of experiments to investigate the function of CD271^+^CD56^+^ BMSC-Exos, which demonstrated their uptake by neurons in the damaged area after SCI, significantly promoting axon regeneration and functional recovery.

The rapid clearance and easy destruction of exosomes limit their therapeutic efficacies 44. Therefore, combining exosomes with biomaterials to achieve long-term release of exosomes without interfering with their biological functions has become a research hotspot in biomedical engineering. GelMA is a hydrogel with porous structure 45. After UV irradiation in the presence of LAP, it can be polymerized through free radical reaction, and effectively preserve drug factors and extend the half-life of drug release. In view of the advantages of GelMA such as plasticity, biocompatibility and biodegradability 46, we prepared the Exos hydrogel sustained-release system and conducted a series of characterization. We found that it has good biocompatibility and simulates the mechanical properties of natural spinal cord. After implantation in vivo, it achieves local continuous delivery of extracellular vesicles at the site of SCI. Meanwhile, this study is a beneficial attempt to improve the administration of exosomes, providing new prospects for the treatment of SCI.

Exosomes contain various substances, which can be transferred to target cells through intercellular communication. MicroRNAs loaded in exosomes play a crucial role in regulating biological processes 47. In spinal motor neurons, miRNAs act as important regulators of development, differentiation, synaptic morphology, and axonal growth 48. Overexpression of miR-431 in damaged neurons enhances axon regeneration of the growth cone 49. MiR-431 was initially identified as a central nervous system-specific miRNA due to its high expression in the brain and spinal cord during embryonic development 50. Upregulation of miR-431 was observed in DRG neurons after injury, stimulating axon regeneration. MiR-431 regulates Wnt signal transduction, which is crucial for neurogenesis and axonal growth, by reducing the expression of the Wnt antagonist Kremen1 51. Furthermore, miR-431 prevents synaptic loss in an induced Alzheimer's disease neuronal cell culture model by silencing Kremen1β 52. In our study, we performed microRNA sequencing to identify highly expressed microRNA components in CD271^+^CD56^+^ BMSC-Exos. Our results indicated abundant levels of miR-431-3p in CD271^+^CD56^+^ BMSC-Exos, playing a significant role in promoting axon regeneration and synapse formation. To elucidate the effect of miR-431-3p in CD271^+^CD56^+^BMSC-Exos, we utilized miR-431-3p inhibitors to downregulate its expression. The reduction in miR-431-3p expression in CD271^+^CD56^+^ BMSC-Exos resulted in a diminished positive effect on SCI treatment, highlighting the critical role of miR-431-3p as a key component mediating the promotion of functional recovery. In vitro experiments demonstrated that miR-431-3p mimics alone could not fully replicate the effectiveness of CD271^+^CD56^+^ BMSC-Exos, suggesting that the impact of exosomes is likely due to the presence of multiple bioactive molecules rather than a single microRNA. Therefore, further research is warranted to investigate whether CD271^+^CD56^+^ BMSC-Exos enhance SCI repair through other factors.

RGMA is a glycosylphosphatidylinositol-linked glycoprotein initially identified as an inhibitory guidance cue for axon projection 53, 54. RGMA binds to its receptor neogenin, leading to the inhibition of neurite growth. RGMA is associated with various central nervous system diseases and is upregulated around the lesion site of spinal cord injuries 55. Treatment with RGMA neutralizing antibodies at the injury site in SCI animal models significantly enhances axon regeneration and motor function recovery 56. This antibody's effect is thought to be dependent on the inhibition of the RhoA and ROCK signaling pathways, which are induced by RGMA and inhibit axonal growth 56, 57. Moreover, RGMA inhibition has been shown to enhance corticospinal tract synapse formation after SCI 56, 58, 59. Our study reveals that miR-431-3p from CD271^+^CD56^+^ BMSC-Exos downregulates RGMA expression. Luciferase reporter gene analysis confirmed RGMA as a direct target gene of miR-431-3p. Additionally, in vitro functional tests on DRG confirmed that miR-431-3p from CD271^+^CD56^+^ BMSC-Exos promotes axon regeneration and synapse formation by targeting RGMA. These findings suggest that CD271^+^CD56^+^ BMSC-Exos may represent a potential therapeutic approach targeting RGMA.

In conclusion, our study utilized single-cell RNA sequencing analysis to identify a unique subpopulation of CD271^+^CD56^+^ BMSCs with axon regeneration-promoting function. CD271^+^CD56^+^ BMSC-Exos can transfer miR-431-3p to neurons, thereby inhibiting RGMA expression and promoting axon regeneration and functional recovery after SCI. Exos derived from CD271^+^CD56^+^ BMSCs show potential as a therapeutic approach for SCI, although further research is necessary before their clinical application. Firstly, the subdivision of BMSCs into functional subpopulations based on cell surface markers is limited due to overlapping expression within subpopulations, necessitating standardized functional characterization of potential BMSC subpopulations. Secondly, the isolation of specific BMSC subpopulations for exosome research may result in the loss of some BMSC characteristics. Future research should focus on developing a process for isolating BMSC subpopulations and extracting exosomes while preserving their natural markers and functions.

## Supplementary Material

Supplementary figures and tables.

## Abbreviations

BMS: basso mouse scale
BMSCs: bone marrow-derived MSCs
DIR: 1,1-octadecyl-3,3,3-tetramethylindole tricarbonylcyanine iodide
DRG: dorsal root ganglia
FBS: fetal bovine serum
MEM: minimum essential medium
MEPs: motion-evoked potentials
RGMA: repulsive guidance molecule family member a
SCI: spinal cord injury
scRNA-seq: single-cell RNA sequencing
SEM: scanning electron microscope
TEM: transmission electron microscopy
