# Supplementary material for: Exosomes derived from CD271+CD56+ bone marrow mesenchymal stem cell subpopoulation identified by single-cell RNA sequencing promote axon regeneration after spinal cord injury
Source: Theranostics. 2024 Jan 1;14(2):510–27. doi: 10.7150/thno.89008 (PMC10758065; doi:10.7150/thno.89008)
Supplement: Supplementary file 1 — Supplementary figures and tables. [file thnov14p0510s1.pdf]

Supplementary materials

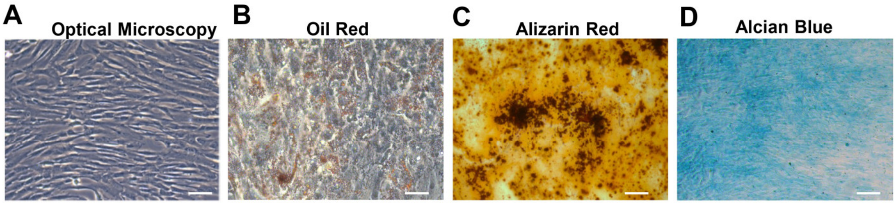

Figure S1. Multilineage differentiation analysis of CD271⁺CD56⁺ BMSCs.

(A) The morphology of $CD271^+CD56^+$ BMSCs. Scale bar, 30 μm. **(B-D)** $CD271^+CD56^+$ BMSCs demonstrated osteogenic, adipogenic and chondrogenic pluripotent differentiation abilities. Scale bar, 30 μm.

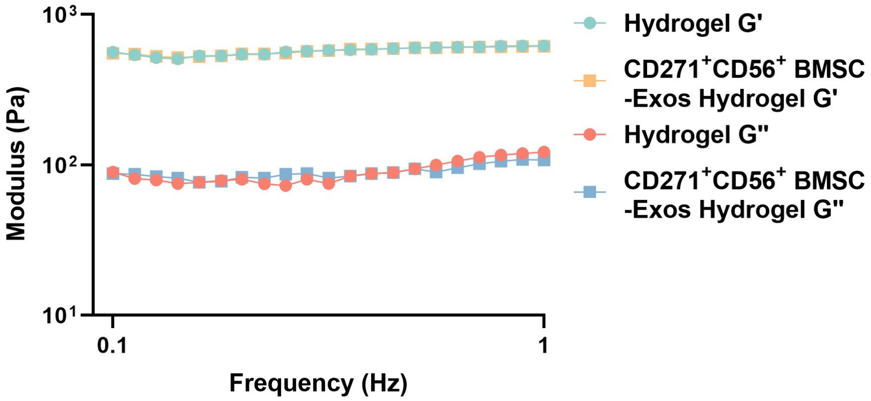

Figure S2. The rheological properties of the hydrogel.

Storage and loss modulus of hydrogels with and without the loading of $CD271^+CD56^+$ BMSC-Exos.

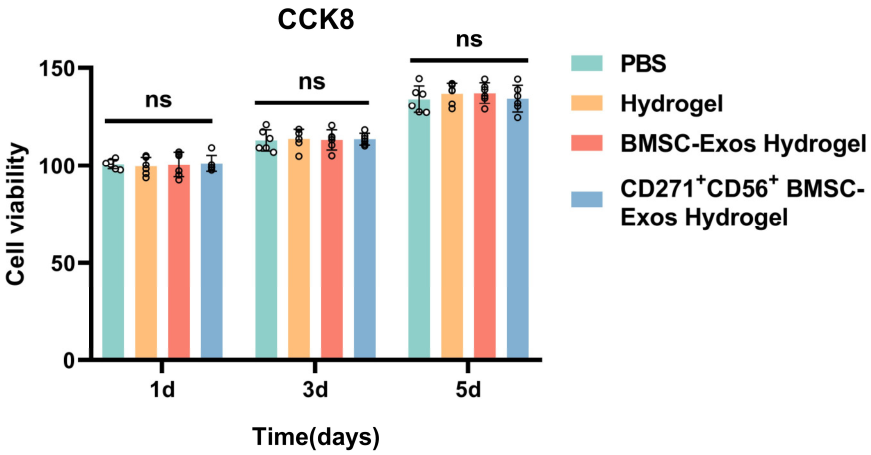

Figure S3. Biocompatibility analysis with NSCs using CCK-8 method.

The data was shown as a mean ± SD, * $P < 0.05$, ** $P < 0.01$. (One-way ANOVA between multiple groups plus Tukey's hoc test).

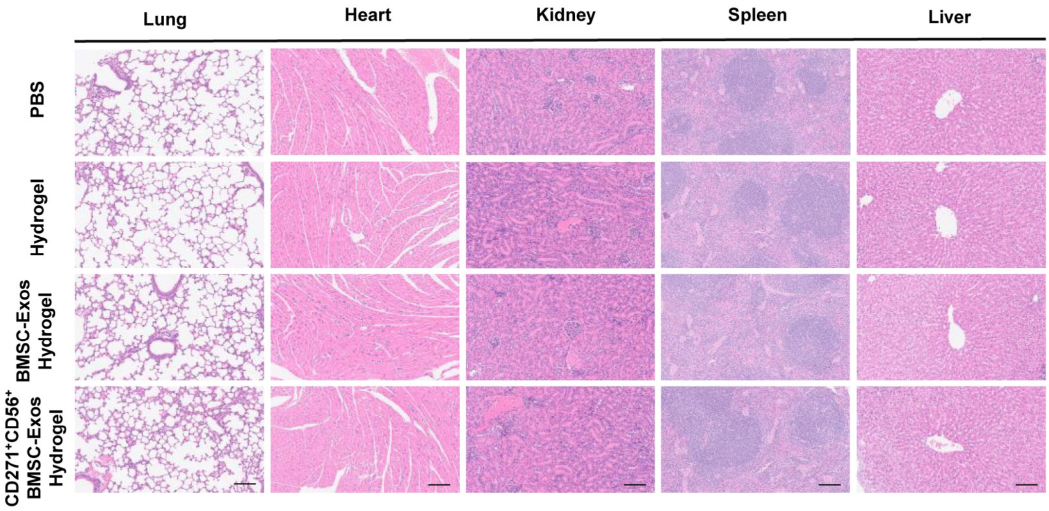

Figure S4. Biocompatibility test in vivo.

Representative images of HE-staining of the heart, liver, spleen, lung, and kidney tissue showed no differences between the PBS, hydrogel, BMSC-Exos hydrogel and CD271^{+}CD56^{+} BMSC-Exos hydrogel treated mice groups. Scale bar, 100 μm.

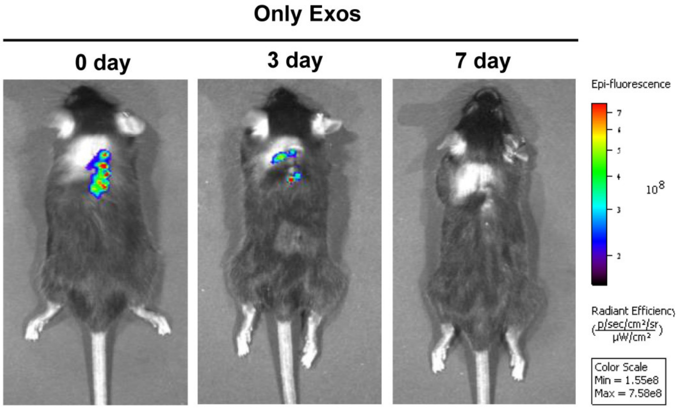

Figure S5. In vivo tracking of DiR-labeled Exos in the injured spinal cord after 0, 3, and 7 days of injury center only exosome administration.

Each group n = 6.

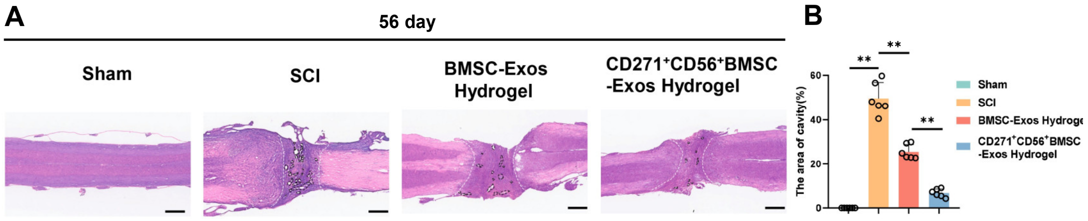

Figure S6. CD271$^+$CD56$^+$ BMSC-Exos hydrogel reduce cavity area after SCI.

(A) H&E staining in each group at 56 days post-SCI. Scale bar, 500 μm. **(B)** Quantification results for cavity area are shown in (A), n = 6. The data was shown as mean ± SD, * P < 0.05, ** P < 0.01. (One-way ANOVA between multiple groups plus Tukey's hoc test).

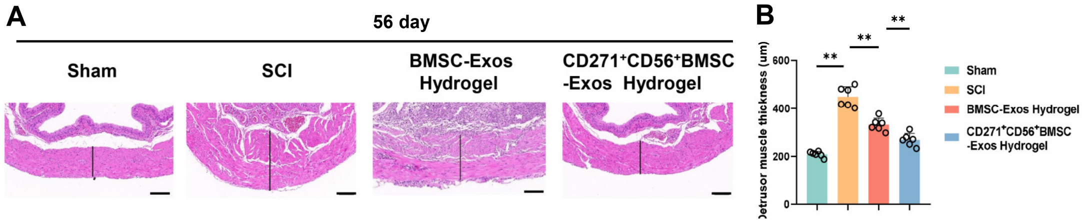

Figure S7. CD271$^+$CD56$^+$ BMSC-Exos hydrogel improve bladder function after SCI

(A) H&E staining of bladder sections from each group at 56 days post-SCI. The black line indicates the detrusor muscle. Scale bar, 100 μm. **(B)** Quantification of detrusor muscle thickness in (A), n = 6. The data was shown as mean ± SD, * P < 0.05, ** P < 0.01. (One-way ANOVA between multiple groups plus Tukey's hoc test).

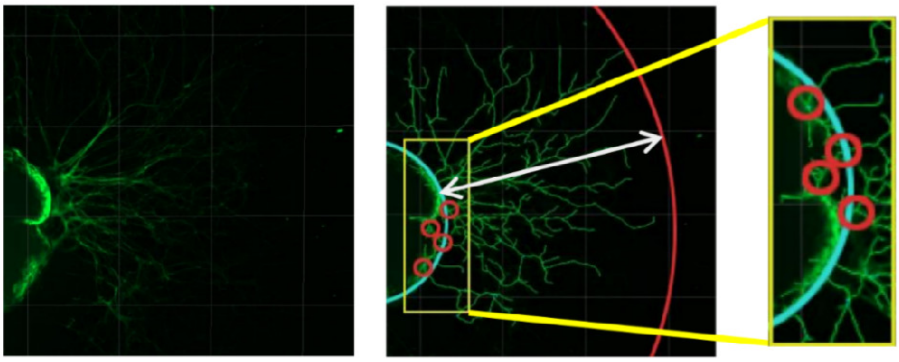

Figure S8. Quantitative schematic of dorsal root ganglion (DRG) axon length and number of branches.

Length quantification was performed using an automated axonal tracing retrieval by Imaris (Oxford

Instruments, UK), within the blue circle is the body of the ganglia, and the pale blue line with two arrows indicates the longest axon extension distance. Intersections (red circles) indicate axon branches.

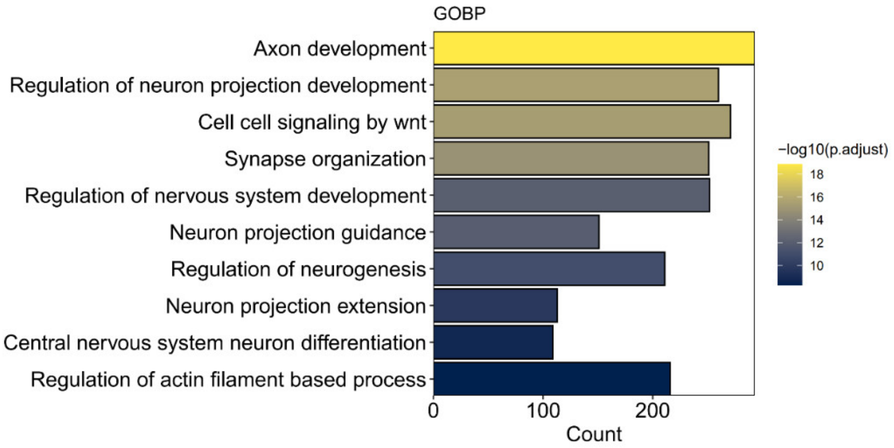

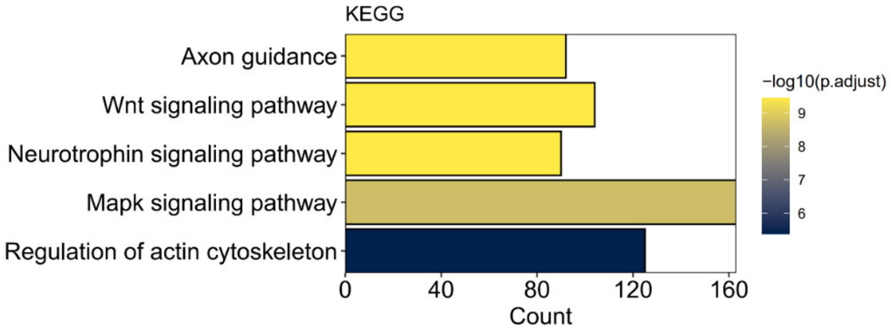

Figure S9. Enriched GO and KEGG terms associated with neural regeneration in CD271$^+$CD56$^+$ BMSC-Exos.

Bar chart shows the number of genes enriched in each term. Color indicates the adjust p values.

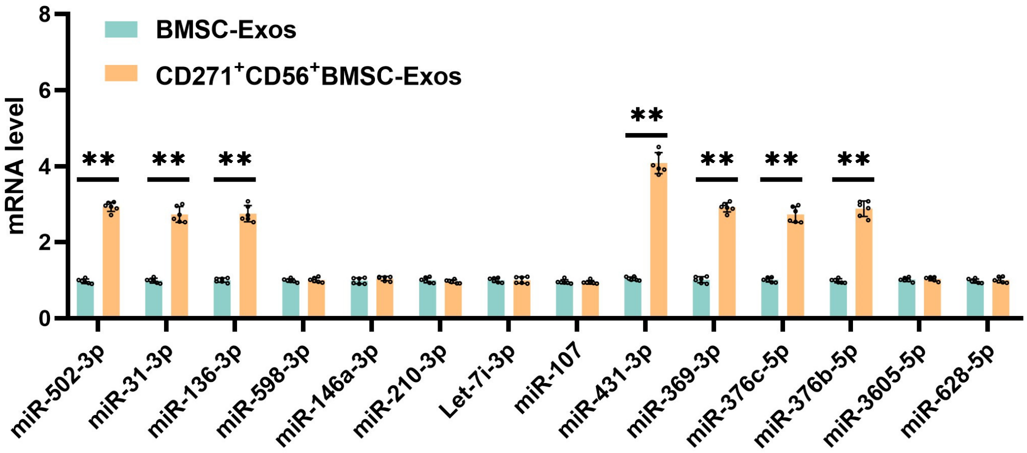

Figure S10. Quantification of the top 14 significantly expressed miRNAs between BMSC-Exos and CD271$^+$CD56$^+$ BMSC-Exos by qRT-PCR analysis.

Each group n = 6. ** P < 0.01 (Two-tailed Student's t-test among two groups).

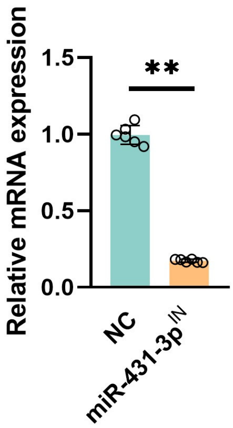

Figure S11. The efficiency of miR-431-3p inhibitor on CD271$^+$CD56$^+$ BMSCs.

Quantitative PCR analysis of miR-431-3p in CD271$^+$CD56$^+$ BMSCs treated with miR-431-3p NC-inhibitor or inhibitor. Each group n = 6. ** P < 0.01 (Two-tailed Student's t-test among two groups).

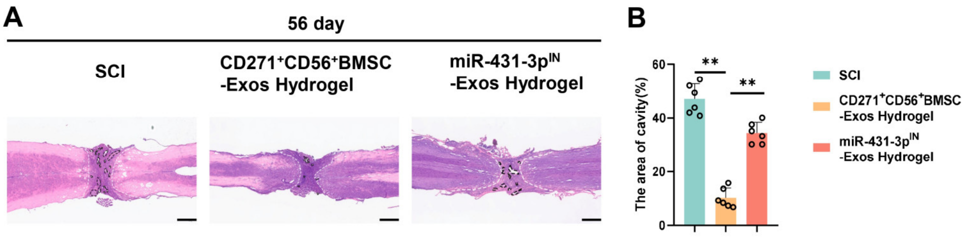

Figure S12. miR-431-3p mediates the effect of CD271$^+$CD56$^+$BMSC-Exos on reducing cavity area after SCI

(A) H&E staining in each group at 56 days post-SCI. Scale bar, 500 μm. **(B)** Quantification results for cavity area are shown in (A), n = 6. The data was shown as mean ± SD, * P < 0.05, ** P < 0.01. (One-way ANOVA between multiple groups plus Tukey's hoc test).

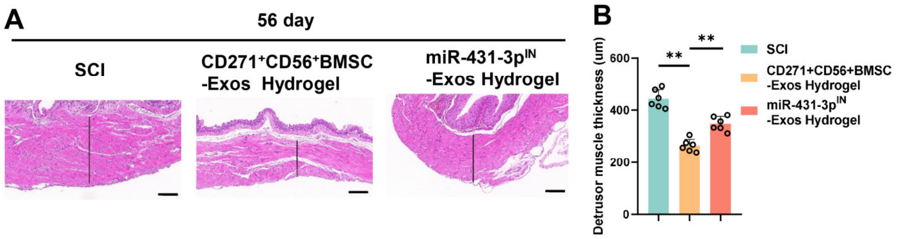

Figure S13. miR-431-3p mediates the effect of CD271$^+$CD56$^+$BMSC-Exos hydrogel on improving bladder function after SCI

(A) H&E staining of bladder sections from each group at 56 days post-SCI. The black line indicates the detrusor muscle. Scale bar, 100 µm. **(B)** Quantification of detrusor muscle thickness in (A), n = 6. The data was shown as mean ± SD, * $P < 0.05$, ** $P < 0.01$. (One-way ANOVA between multiple groups plus Tukey's hoc test).

Table S1. Antibody catalog

IF antibody	company	catalog number	dilution
rabbit anti-NF	CellSignalingTechnology	30564	1:400
goat anti-GFAP	Abcam	Ab53554	1:400
rabbit anti-NeuN	CellSignalingTechnology	12943	1:400
rabbit anti-RGMA	Proteintech	12387-1-AP	1:100
mouse anti- Synaptophysin	Abcam	ab8049	1:200
**WB antibody**	**company**	**catalog number**	**dilution**
rabbit anti-CD9	Proteintech	20597-1-AP	1:1000
rabbit anti-CD63	Proteintech	25682-1-AP	1:1000
rabbit anti-CD81	Proteintech	27855-1-AP	1:1000
rabbit anti-calnexin	Proteintech	10427-2-AP	1:1000
rabbit anti-GAPDH	Elabscience	E-AB-40337	1:5000
rabbit anti- RGMA	Proteintech	12387-1-AP	1:1000
**Flow cytometry antibody**	**company**	**catalog number**	**dilution**
anti-CD271-APC	Biolegend	345108	1:100
anti-CD31-PE	Biolegend	303106	1:100
anti-CD45-PE	Biolegend	304008	1:100
anti-CD235-PE	Biolegend	306604	1:100
anti-CD11b-PE	Biolegend	101208	1:100
anti-CD90-APC/Cy7	Biolegend	344020	1:100
anti-CD73-PE/CF594	Biolegend	155306	1:100
anti-CD56-PE/Cy7	Biolegend	318318	1:100
Zombie Aqua	Biolegend	77143	1:500

Table S2. All primer sequences used for qRT-PCR.

RGMA	Forward primer	ATGGACGAACTTCCGTCTGC
	Reverse primer	ACGGCGTTGACTACCTCCT
GAPDH	Forward primer	GTGGCAAAGTGGAGATTGTTG
	Reverse primer	CGTTGAATTTGCCGTGAGTG
miR-628-5p	Forward primer	GCCGATGCTGACATATTTACTAGAGG
miR-3605-5p	Forward primer	TGAGGATGGATAGCAAGGAAGCC
miR-376b-5p	Forward primer	GCCCGTGGATATTCCTTCTATGTTT
miR-376c-5p	Forward primer	CGGCGGTGGATATTCCTTCTATGTT
miR-369-3p	Forward primer	GCCGGCGAATAATACATGGTTGATC
miR-431-3p	Forward primer	CAGGTCGTCTTGCAGGGCTT
miR-107	Forward primer	AGCAGCATTGTACAGGGCTATCA
Let-7i-3p	Forward primer	CTGCGCAAGCTACTGCCTT
miR-210-3p	Forward primer	TATTCTGTGCGTGTGACAGCG
miR-146a-3p	Forward primer	GGCCCTCTGAAATTCAGTTCTTCAG
miR-598-3p	Forward primer	CGTACGTCATCGTTGTCATCGTCA
miR-136-3p	Forward primer	CGTGCTATGCCAACATATTGCCAT
miR-31-3p	Forward primer	GCTGCTATGCCAACATATTGCCAT
miR-502-3p	Forward primer	AATGCACCTGGGCAAGGATTCA
Universal U6 Primer F (Sangon biotech, China)	-	-
Universal PCR Primer R (Sangon biotech, China)	-	-
